# Continuum descriptions of cytoskeletal dynamics

**DOI:** 10.1186/1477-3155-11-S1-S5

**Published:** 2013-12-10

**Authors:** Karsten Kruse

**Affiliations:** 1 Theoretische Physik, Universität des Saarlandes, Postfach 151150, 66041 Saarbrücken, Germany

## Abstract

This tutorial presents an introduction into continuum descriptions of cytoskeletal dynamics. In contrast to discrete models in which each molecule keeps its identity, such descriptions are given in terms of averaged quantities per unit volume like the number density of a certain molecule. Starting with a discrete description for the assembly dynamics of cytoskeletal filaments, we derive the continuity equation, which serves as the basis of many continuum theories. We illustrate the use of this approach with an investigation of spontaneous cytoskeletal polymerization waves. Such waves have by now been observed in various cell types and might help to orchestrate cytoskeletal dynamics during cell spreading and locomotion. Our analysis shows how processes at the scale of single molecules, namely, the nucleation of new filaments and filament treadmilling, can lead to the spontaneous appearance of coherent traveling waves on scales spanning many filament lengths. For readers less familiar with calculus, we include an informal introduction to the Taylor expansion.

## Introduction

With the advancements in microscopy techniques it has become increasingly clear that a true understanding of many cellular phenomena requires to take spatial aspects into account. This is clearly the case for the cytoskeleton as illustrated by the changes in the microtubule network during cell division or the reorganization of the actin meshwork during cell locomotion. To reach a quantitative understanding of the mechanisms underlying these processes, concepts and methods from physics can be extremely valuable. These methods include notably the theoretical analysis of cellular systems. Most biological and medical curricula today lack, unfortunately, a thorough introduction into mathematical and physical tools, which often leads to discomfort on the part of biologists and physicians, when confronted with the results of a theoretical study. This holds in particular for continuum descriptions. This tutorial is meant to familiarize life scientists with the basic ideas underlying this approach.

Continuum theories have had a great success in describing static and dynamic phenomena for large classes of matter. Well-known examples range from simple fluids [1] and elastic materials [2] to fluid membranes and vesicles [3]. Less conventional examples include the cytoskeleton [4] and even flocks of birds [5]. Especially for flocks of birds, one might at first sight be inclined to use a discrete approach instead, in which the position and behavior of each individual bird is considered. Such models indeed exist and have yielded valuable insights, see for example [6]. Similarly, simulation tools like cytosim allow the user to study cytoskeletal dynamics, while keeping track of each individual cytoskeletal filament, of each motor molecule, and of any other cytoskeletal protein possibly present, for example, the passive cross-linker *α*-actinin [7]. There are, however, two major problems associated with this approach. One is most easily revealed by considering water. In a discrete description, we would need to track the position of each individual water molecule, its momentum, and its interaction with all surrounding water molecules. Given the astronomic number of water molecules even in small volumes, this is a daunting task. Furthermore, one needs to provide details of the interactions between the individual entities and it is a priori not clear, which of these details will be important for the large scale dynamics of a system.

In contrast, in a continuum theory, the ultimately particulate nature of a material or system is neglected. Intuitively, this approximation is valid for structures on length scales that are very much larger than the discrete constituents of the material under investigation. Maybe somewhat surprisingly, though, they often describe a material's behavior correctly even down to length scales that approach the constituents' sizes. The Navier-Stokes equation, for example, describes the flow of water in micro- and to some extent even in nanofluidic devices [8]. From a more technical point of view, continuum theories often have the advantage over more microscopic descriptions to provide a comprehensive account of possible material behavior. Furthermore, many of the details of the interactions between the constituents are lumped together into a comparatively small set of parameters. In this tutorial, we show how continuum theories can be developed and used in a cellular context to describe cytoskeletal dynamics.

Our aim is not to enable you to formulate and solve your own continuum theories. Still, you should get a feeling for the method and for what you can and cannot expect from this approach. This tutorial necessarily contains a certain amount of mathematics. To make the tutorial accessible to an audience not using calculus on an everyday basis, we have tried to make all steps in the calculations explicit. You should have a pencil and a sheet of paper ready to follow them in detail. In addition, some calculations are left for the reader. To perform them you do not need much beyond a sincere interest in these techniques and some basic knowledge of calculus, notably to take derivatives. To keep the mathematics tractable, we have also simplified certain biological processes. The emphasis of this tutorial is really on mathematical and physical concepts rather than on a detailed analysis of cellular processes.

The tutorial is organized as follows. We start with a discrete description of the length dynamics of cytoskeletal filaments from which we derive a continuum theory. This approach will lead us to the continuity equation. We will use this equation to develop a continuum description of treadmilling filaments interacting with nucleation promoting factors. We will use this formalism to study a possible mechanism underlying spontaneous polymerization waves. Two excursions aim at recalling the Taylor expansion and give a general account of the continuity equation.

### Mathematical prelude: The Taylor expansion

A mathematical tool that we will make frequent use of when going to the continuum limit is the so-called Taylor expansion of a (real-valued) function. The aim here is not to give a mathematically rigorous account of the Taylor expansion, but rather to introduce the basic idea. Consequently, we assume that the functions we will deal with are all sufficiently well-behaved such that all operations we perform on them are allowed.

**Figure 1:**
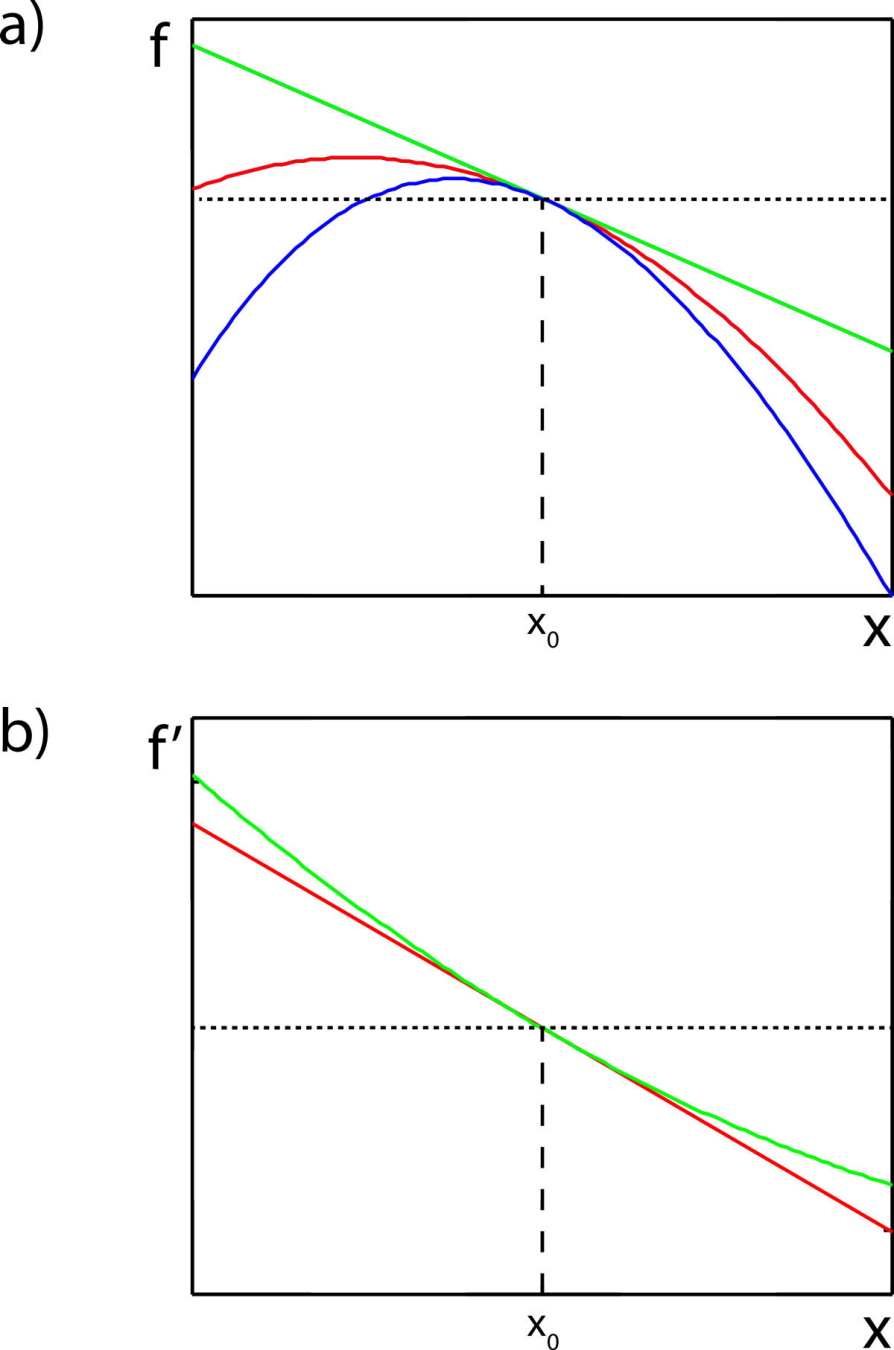
**The Taylor expansion**. **a) **Various polynomial approximations to a function *f *in the vicinity of *x *= *x*_0 _(blue line). The simplest approximation is to use the value *f*(*x*_0_) (dotted line). The "first-order approximation" is given by the tangent to *f *in *x *= *x*_0 _(green line). The "second-order approximation" is given by the quadratic polynomial $f\left(x_{0}\right)+f^{\prime }\left(x_{0}\right)\left(x-x_{0}\right)+\frac{1}{2}f^{\prime \prime }\left(x_{0}\right){\left(x-x_{0}\right)}^{2}$ (red line). **b) **Polynomial approximations to the derivative *f*' of *f *in the vicinity of *x *= *x*_0 _(green line). The tangent to *f*' in *x *= *x*_0 _is given by the derivative of the second-order approximation to *f *(red line).

The aim of the Taylor expansion is to approximate a function in the vicinity of a chosen point by a polynomial, usually of low order. Consider the function *f *displayed on Figure 1a and the point *x*_0_. The value of the function at *x*_0 _is denoted by *f *(*x*_0_). A very crude approximation of *f *in the vicinity of *x*_0 _is *f*(*x*_0_). An obviously better approximation of *f *is given by the tangent to *f *in *x*_0_. If we denote the slope of *f *in *x*_0 _by *f*'(*x*_0_), then the tangent is given by the expression *f*(*x*_0_) + *f*'(*x*_0_)(*x - x*_0_), where the slope of *f *in *x*_0 _is just the derivative of *f *in *x*_0_, that is,

(1) $$f^{\prime }=\frac{\text{d}f}{\text{d}x}.$$

Here, we have suppressed the dependence on *x*_0 _for the ease of notation.

Now, the derivative of a function is itself a function. If we use the tangent to approximate the function *f*, then this amounts to the same as to approximating the derivative by a constant, namely *f*'(*x*_0_), see Figure 1b. As above, we can improve the approximation by using the tangent to *f*' at *x*_0_. Denoting the slope of the derivative in *x*_0 _by *f*"(*x*_0_), we can write *f*'(*x*) *≈ f*'(*x*_0_) + *f*"(*x*_0_)(*x - x*_0_). To be consistent, we should also modify our approximation for *f*. Putting

(2) $$f\left(x\right)\approx f\left(x_{0}\right)+f^{\prime }\left(x_{0}\right)\left(x-x_{0}\right)+\frac{1}{2}f^{\prime \prime }\left(x_{0}\right){\left(x-x_{0}\right)}^{2},$$

we see that the derivative of the approximation for *f *equals the approximation for *f*'. The degree of the polynomial used to approximate a function *f *is called the *order *of the approximation.

**Your turn: **Calculate the Taylor expansion of the exponential function exp(*x*) with respect to *x*_0 _= 0 up to second order.

Obviously, this scheme can be iterated further to get better and better approximations of *f *in the vicinity of *x*_0_. However, we will content ourselves in the following with approximations up to second order at most.

### Length-dynamics of active polar filaments - part I

As a first example for a continuum description of cytoskeletal dynamics, we will consider the assembly and disassembly of cytoskeletal filaments. Actin filaments and microtubules are linear aggregates of non-covalently bound subunits, namely G-actin and tubulin dimers, respectively. G-actin is a molecule of about 42 kDa with linear extensions of about 6.7 nm and 4 nm, whereas a tubulin dimer has a molecular weight of about 110 kDa and is about 8 nm long and 4 nm in diameter. The length distributions of F-actin and of microtubules in cells is currently largely unknown, but typical values have been estimated to be around 500 nm up to a few microns for F-actin and up to several tens of microns for microtubules. These subunits are structurally polar resulting in *polar *filaments with different subunit attachment and removal rates at the two ends. For further reference, we will call the fast growing end the plus-end and the other end the minus-end. In addition, G-actin and tubulin can bind nucleotides and their rates of attachment to and removal from a filament depend on the state of the nucleotide bound, that is, whether it is a nucleoside triphosphate (NTP) or a nucleoside diphosphate (NDP). Through hydrolysis of an NTP energy can be introduced into the system on a molecular level. This is the defining property of an *active *material.

The structural polarity together with the activity makes the assembly and disassembly dynamics of cytoskeletal filaments more interesting than that of most polymers that are usually studied. In particular, it can lead to a phenomenon called treadmilling, where the plus-end grows on average, while the minus-end shrinks on average [9,10]. This is the situation we will consider in the following without further questioning the origin of this phenomenon. Also, we will consider generic aspects of treadmilling filaments rather than specifically actin filaments or microtubules, because here we are mostly interested in introducing the concept of continuum theories.

We consider the situation of active polar filaments that assemble in a solution containing subunits. Let *c*_*L *_denote the concentration of filaments consisting of *L *= 1, 2, ... subunits. For now, we assume that the filaments are homogeneously distributed and will consider a possible space-dependence of the distribution later. The time evolution of *c_L _*is given by

(3) $$\frac{d}{dt}c_{L}=k_{a}c_{L-1}-k_{a}c_{L}-k_{d}c_{L}+k_{d}c_{L+1},$$

where *k_a _*and *k_d _*are the rates of subunit attachment at the plus- and of subunit removal at the minus-end, respectively. These rates depend strongly on the environmental conditions and on the state of the nucleoside bound, but typical values are about 10s^-1^*μ*M^-1 ^for the attachment rate of ATP-G-actin at the plus-end of an actin filament and 0.25s^-1 ^for the removal rate of ADP-G-actin at the minus-end [11]. Note, however, that the effective rates of subunit addition at the plus- and subunit removal at the minus-end result from complex dynamic processes inside the filament, such that they can strongly deviate from these values. For the time being, we will consider them to be constant, which amounts to assuming a constant reservoir of filament subunits. We will discuss a possible experimental realization below.

While there is in principle nothing wrong with this discrete description, it turns out that a continuum description is easier to analyze. The idea is to neglect the discrete nature of the subunits and consider a continuous length distribution. Indeed, if you are interested in the behavior of the length distribution on scales that are larger than many subunit sizes, many details are irrelevant.

Technically, we start by replacing the concentrations *c_L _*by a *density c*(*ℓ*), such that *c_L _*= *c*(*Lδ*)*δ*, where *δ *is the change in filament length upon addition or removal of a subunit. We thus have

(4) $$\frac{d}{dt}c\left(L\delta \right)=k_{a}c\left(\left(L-1\right)\delta \right)-k_{a}c\left(L\delta \right)-k_{d}c\left(L\delta \right)+k_{d}c\left(\left(L+1\right)\delta \right).$$

If we are interested in structures that are significantly larger than *δ*, we can treat *δ *as a small quantity. Consequently, we can use the Taylor expansion to approximate *c*((*L *± 1)*δ*) by a linear function:

(5) $$c(\left(L\pm 1\right)\delta \approx c\left(L\delta \right)\pm c^{\prime }\left(L\delta \right)\delta .$$

Inserting this expression in Eq. (4) we obtain

(6) $$\frac{d}{dt}c\left(L\delta \right)=k_{a}\left[c\left(L\delta \right)-c^{\prime }\left(L\delta \right)\delta \right]-k_{a}c\left(L\delta \right)-k_{d}c\left(L\delta \right)+k_{d}\left[c\left(L\delta \right)+c^{\prime }\left(L\delta \right)\delta \right]$$

(7) $$=-k_{a}c^{\prime }\left(L\delta \right)\delta +k_{d}c^{\prime }\left(L\delta \right)\delta$$

The important step is now to replace the discrete expression *Lδ *by the continuous variable *ℓ*. Intuitively, this makes sense as long as you do not consider features of the distribution *c*(*ℓ*) on length scales that are of the order of the subunit size or smaller. It also implies that molecular details of the assembly processes can be irrelevant for the large scale structures. We thus arrive at

(8) $$\frac{\partial }{\partial t}c\left(\ell ,t\right)\;\;=\;\;-\left(v_{a}-v_{d}\right)\frac{\partial }{\partial \ell }c\left(\ell ,t\right).$$

Here, *v_a _*= *k_a_δ *and *v_d _*= *k_d_δ *are the assembly and disassembly velocities. Furthermore, in this expression the derivative with respect to time, *d/dt *has been replaced by a *partial derivative ∂/∂t*. The reason is that the density *c *depends on two continuous variables, *ℓ *and *t*, and taking the partial derivative indicates that we want to calculate the derivative with respect to *t *for fixed *ℓ*. Analogously, *∂/∂ℓ *indicates that we take the derivative with respect to *ℓ *for fixed *t*.

### Interlude: The continuity equation

The partial differential equation that we derived for the length dynamics of treadmilling filaments can be cast into the following form

(9) $$\frac{\partial }{\partial t}c\left(\ell ,t\right)=-\frac{\partial }{\partial \ell }j,$$

where *j*(*ℓ*, *t*) = (*v_a _- v_d_*)*c*(*ℓ*, *t*). Such an equation is known as a *continuity equation*. It generally describes the evolution of a conserved quantity with *j *being the associated current. We will now see, how such an equation can be obtained on general grounds.

**Figure 2:**
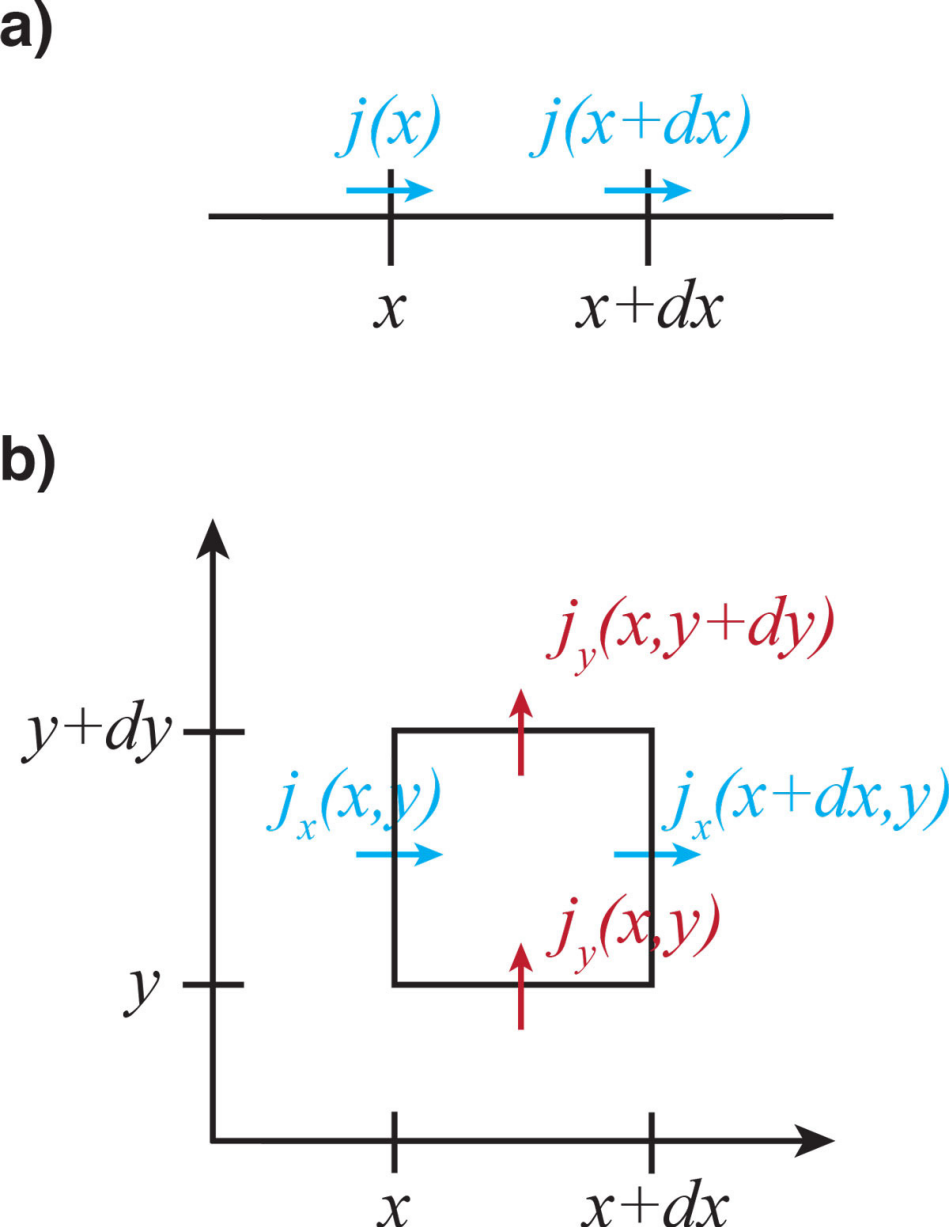
**The continuity equation**. **a) **Flux balance in one dimension: the quantity  Q can enter or leave the interval [*x*, *x *+ Δ*x*] only via flux *j *through the boundaries. **b) **Flux balance in two dimensions: the quantity  Q can enter or leave the rectangular region of size Δ*x *× Δ*y *only via flux **j **through the boundaries.

For simplicity, we start with a one-dimensional system and will then consider the general case. Consider a quantity  Q that is distributed along an axis. This could, for example, be the electric charge along a DNA molecule. Let's denote by *Q*(*x*) the amount of  Q in the intervall [*x*, *x *+ Δ*x*] and assume that  Q can neither be generated nor destroyed. In that case *Q*(*x*) can only change through a flux of  Q across the boundaries of the interval. Let's denote this flux by *j*, which by convention is positive if directed towards larger *x*, see Figure 2a. Formally, we then have the flux balance equation

(10) $$\frac{d}{dt}Q\left(x\right)=j\left(x\right)-j\left(x+\Delta x\right).$$

Using the same expansion as above, we find

(11) $$\frac{d}{dt}Q\approx j\left(x\right)-\left[j\left(x\right)+\frac{\partial }{\partial x}j\left(x\right)\Delta x\right]$$

(12) $$=-\frac{d}{dx}j\left(x\right)\Delta x.$$

If we divide by Δ*x *and consider the limit Δ*x → *0, then we get

(13) $$\frac{\partial }{\partial t}q=-\frac{\partial }{\partial x}j,$$

where *q*(*x*) = lim_Δ*x→*0 _*Q*(*x*)*/*Δ*x *is the (line) density associated with  Q. This is the one-dimensional continuity equation and has the same form as Eq. (9).

If we go to higher dimensions, then the flux becomes a vector **j **with components *j_x_*, *j_y_*, and so on denoting the components of the current in *x*- and *y*-direction, respectively. To calculate the change of a quantity in a small box, one then has to consider the contributions of the current in all directions, see Figure 2b. In two spatial dimensions, this leads to

(14) $$\frac{\partial }{\partial t}q=-\frac{\partial }{\partial x}j_{x}-\frac{\partial }{\partial y}j_{y}.$$

Note, that here *q *is now a surface density.

**Your turn: **Derive Equation (14). How does it look in three spatial dimensions?

The right hand side of Eq. (14) is called the divergence of **j **and is often written as ∇·**j**. Adding a source and sink term $S_{Q}$ to account for possible generation or destruction of  Q, we can write

(15) $$\frac{\partial }{\partial t}q+\nabla \cdot j=S_{Q},$$

which is the most general form of the continuity equation.

Example: A reaction-diffusion system

As a specific example consider the diffusion of two molecular species A and B that can be converted into each other. For specificity you might have a protein in mind that can change between two different conformations. We describe the distribution of the molecules by the number densities *a *and *b*, respectively. The continuity equations for the two molecular species then read:

(16) $$\frac{\partial }{\partial t}a+\nabla \cdot j_{\text{a}}=S$$

(17) $$\frac{\partial }{\partial t}b+\nabla \cdot j_{\text{b}}=-S.$$

The molecules are assumed to diffuse in an aqueous solution, such that their currents are directed along the corresponding concentration gradients. Explicitly, the components of the currents are (in three dimensions)

(18) $$j_{\text{a},x}=-D_{\text{a}}\frac{\partial }{\partial x}a$$

(19) $$j_{\text{a},y}=-D_{\text{a}}\frac{\partial }{\partial y}a$$

(20) $$j_{\text{a},z}=-D_{\text{a}}\frac{\partial }{\partial z}a$$

and

(21) $$j_{\text{b},x}=-D_{\text{b}}\frac{\partial }{\partial x}b$$

(22) $$j_{\text{b},y}=-D_{\text{b}}\frac{\partial }{\partial y}b$$

(23) $$j_{\text{b},z}=-D_{\text{b}}\frac{\partial }{\partial z}b.$$

The diffusion constants *D_a _*and *D_b _*are positive and the minus-signs indicate that the flux is directed from high to low concentrations. Since we assume that species A is only generated and destroyed by conversion into B and vice versa, the source terms in Eqs. (16) and (17) have to have opposite signs. Their functional form depends on the molecular details of the conversion processes. If, for example, conversions occur spontaneously at a rate *ω_a _*from A to B and at rate *ω_b _*from B to A, then one would write *S *= *-ω_a_a *+ *ω_b_b*.

Inserting the expressions for the currents and the source terms and shifting the terms containing the currents to the right hand side, we then get

(24) $$\frac{\partial }{\partial t}a=D_{a}\Delta a-\omega_{a}a+\omega_{b}b$$

(25) $$\frac{\partial }{\partial t}b\;\;=\;\;D_{b}\Delta b+\omega_{a}a-\omega_{b}b.$$

Here, Δ is the so-called Laplace operator. In absence of the source terms, each of the two equations is the common diffusion equation. In presence of the source terms, the two equations define a simple reaction-diffusion system as introduced by A. Turing [12].

**Your turn: **Show that the Laplace operator can be written as

(26) $$\Delta =\frac{\partial^{2}}{\partial x^{2}}+\frac{\partial^{2}}{\partial y^{2}}+\frac{\partial^{2}}{\partial z^{2}}.$$

Boundary conditions

To complete the description of the evolution of  Q in a finite domain one has to specify so-called *boundary conditions*. They determine the behavior of the current and/or the density at the domain boundaries. For example, if you consider particles in a vessel with impenetrable walls, then the current through these walls must vanish, **j**_⊥ _= 0, where **j**_⊥ _denotes the component of the current perpendicular to the wall.

### Length-dynamics of active polar filaments - part II

Let us return to the length distribution of treadmilling filaments and focus on the case that *v_a _> v_d _*such that filaments grow on average. If *v_a _*and *v_d _*are constants, then filament growth would be unbounded. In a real system, though, the pool of subunits is exhausted at some point, which leads to a stop of filament growth. We will consider here a formally simpler alternative, namely that filaments vanish at a constant rate *ν_d _*independent of their length. To our knowledge, there is no experimental evidence for such disassembly of neither actin filaments nor microtubules. However, a more realistic description would at this point only distract from our main goal which is to introduce the idea of continuum theories. One could, however, realize such a dynamics in vitro by removing in regular intervals let's say half of the solution containing the filaments and subsequently adding new buffer containing filament subunits. We neglect possible breaking of filaments during this process as well as filament annealing.

Since partitioning and distributing filaments results in diluting them, we also need to provide a source of new filaments. At physiological monomer concentrations, the rate at which new actin filaments form spontaneously is negligible. Instead cells rely on proteins that assist the formation of new filaments, so-called nucleation promoting factors (NPFs). Common examples of such proteins are the Arp2/3 complex, which when itself bound to an existing filaments generates a new filament that has its pointed end connected to the complex, or members of the formin family, which stay attached to the barbed end of a newly generated filament and assist further elongation. In the following, we will neglect these aspects of NPFs and just consider them to generate new filaments.

Let us assume that we maintain a constant density of NPFs that generate new filaments at a rate *ν*. We can account for this feature by a boundary on the filament current *j *at filament length *ℓ *= 0, that is *j*|_*ℓ *= 0 _= *ν*. The length dynamics of polar filaments is then completely specified by the following two equations:

(27) $$\frac{\partial }{\partial t}c+\frac{\partial }{\partial \ell }\left(v_{a}-v_{d}\right)c=-\nu_{d}c$$

(28) $$\left(v_{a}-v_{d}\right)c\left(\ell =0\right)=\nu .$$

From these equations, we can get the distribution of filament lengths in steady state, that is, for *∂c/∂t *= 0. Explicitly,

(29) $$c_{s}\left(\ell \right)=\frac{\nu }{v_{a}-v_{d}}\exp\left\{-\ell /\lambda \right\}$$

where *λ *= (*v_a _- v_d_*)/*ν_d _*is a characteristic length.

**Your turn: **Show that the distribution *c_s _*of Eq. (29) indeed solves Eq. (27) with *∂c/∂t *= 0 and fulfills the boundary condition Eq. (28).

In the present example, we have derived the continuum theory from an underlying discrete microscopic picture. In general, different microscopic pictures will lead to the same continuum theory. An alternative possibility is to start directly with the continuity equation. In that case, the definition of the current and the source terms reflect essential features of the physics underlying the molecular processes of interest. We will give now an example of this approach and treat the case of spatially heterogeneous distributions of treadmilling filaments interacting with NPFs.

### Theoretical description of a network of filaments

**Figure 3:**
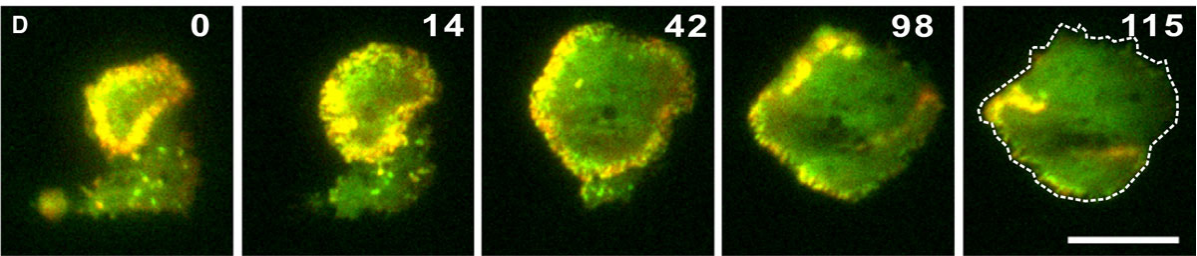
**Example of an actin polymerization wave in Dictyostelium discoideum**. Circular wave expanding the cell border of *Dictyostelium discoideum*. Cells expressing mRFP-LimED to label filamentous actin structures and GFP-Arp3 for incorporation into the Arp2/3 complex were recorded by dual-emission TIRF microscopy during recovery from latrunculin A treatment. The protrusion stops when the wave starts to collapse. Time is indicated in seconds. Scale bar: 10 *μ*m. From Bretschneider et al. [20] with permission from Biophysical Journal.

In recent years, actin waves have been observed in various organisms [13]. These waves appeared either after drug treatment or spontaneously, see Figure 3 for an example. It has been suggested that such waves might orchestrate the cytoskeletal components for cell migration [14,15]. We will now show that the interplay of dynamic polar filaments and nucleation promoting factors (NPFs) can lead to the spontaneous emergence of waves [14,16-18]. The tool we will use in this investigation is again a continuum theory.

**Figure 4:**
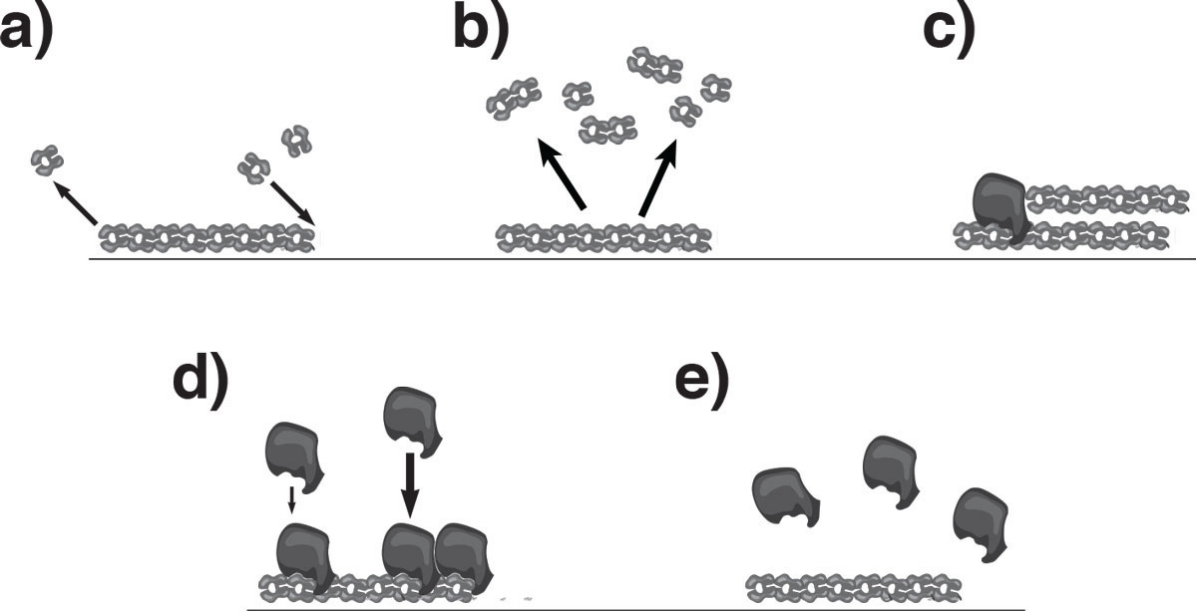
**Schematic representation of filament dynamics in presence of nucleation promoting factors (NPFs)**. **a) **Filaments treadmill by attachment of monomers at one end and detachment at the other. **b) **Filaments disassemble spontaneously. **c) **New filaments are generated by NPFs. **d) **NPFs bind to filaments. Binding is cooperative, favoring attachments in the vicinity of already bound NPFs. **e) **NPFs spontaneously detach from filaments.

To give a full account of the configuration of the actin cytoskeleton, we would need to specify the position, orientation, length, and configuration of the filaments. We will simplify the description by considering only length scales larger than the average filament length and by treating the filaments as points, while retaining the information about their orientation. Furthermore, we will consider a situation, where all filaments are aligned along a single axis as is the case for actin filaments in stress fibers or contractile rings. The processes we intend to capture with our description include filament treadmilling, filament nucleation, and the dynamics of the nucleation promoting factors. They are schematically represented in Figure 4.

Let's turn to the formalism. The state of the filament bundle along the *x*-axis is specified by the densities *c*^+ ^and *c*^- ^of filaments with their plus-end pointing into the direction of increasing and decreasing values of *x*, respectively. The time evolution of *c*^+ ^and *c^- ^*is given by continuity equations:

(30) $$\frac{\partial }{\partial t}c^{+}+\frac{\partial }{\partial x}j_{x}^{+}=S^{+}$$

(31) $$\frac{\partial }{\partial t}c^{-}+\frac{\partial }{\partial x}j_{x}^{-}=S^{-},$$

where $j_{x}^{+}$ and $j_{x}^{-}$ are the respective filament fluxes in the *x*-direction. The source and sink terms *S*^+ ^and *S^- ^*account for the generation of new filaments and their disappearance.

We now specify the expressions for the currents and the source. As above, we assume treadmilling dynamics for the filaments. As a consequence, the filaments will move in space into the direction of the filament, see Figure 4a. In addition, we will account to some extent for fluctuations in the system by introducing an effective diffusion current. This current is proportional to gradients in the filament density and captures thermal fluctuations and to some extent the stochasticity of filament assembly and disassembly. We thus have

(32) $$j_{x}^{+}=vc^{+}-D\frac{\partial }{\partial x}c^{+}$$

(33) $$j_{x}^{-}=-vc^{-}-D\frac{\partial }{\partial x}c^{-},$$

where *v *is the treadmilling velocity. As in the previous section, we also have to introduce boundary conditions. We will use in the following periodic boundary conditions, that is, for a system of size *L*, the densities and currents are *L*-periodic functions, for example, *j*^+^(*x *+ *L*) = *j*^+^(*x*). We use these boundary conditions mostly for computational convenience, but they have also some biological relevance, for example, in the case of contractile actin rings. Let us recall, however, that in this tutorial, we do not want to give a detailed account of biological processes, but rather focus on formal aspects. For a more realistic application of a continuum description of cytoskeletal waves, see for example Refs. [15-18].

The source terms depend on the distribution of nucleating promoting factors (NPFs). The crucial point is, that these have a dynamics on their own, which couples back to the distribution of filaments. Motivated by the Arp2/3 complex, we distinguish two classes of NPFs: bound to filaments and cytosolic. Furthermore, we assume that bound NPFs generate filaments of the same orientation as the filament they are bound to, see Figure 4c. As a consequence, we introduce the densities *n*^+ ^and *n^- ^*of NPFs bound to plus- and minus-filaments, respectively, and the density *n*_c _of cytosolic NPFs. The source terms *S*^+ ^and *S^- ^*then read

(34) $$S^{+}\;\;=\;\;\;\nu \left(n^{+}+\varepsilon n_{c}\right)-\nu_{d}c^{+}$$

(35) $$S^{-}=\;\;\nu \left(n^{-}+\varepsilon n_{c}\right)-\nu_{d}c^{-}.$$

Here, *ν *is the nucleation rate and *ε *a numerical factor that accounts for the weaker nucleation activity of cytosolic NPFs. Finally, similarly to the previous section, *ν_d _*is the rate of filament disassembly, see Figure 4b.

Also the dynamics of the NPFs is governed by continuity equations. The transport of NPFs is purely diffusive. This holds also for bound NPFs as they stay attached to a specific subunit of a filament and as treadmilling does not move filament subunits. Note, however, that the system behavior does not change qualitatively if NPFs are transported with the filaments, as could be the case in presence of molecular motors. For bound NPFs we assume a constant unbinding rate *ω_d_*, see Figure 4e. The attachment dynamics of NPFs to filaments is more involved. First of all there is a base rate *ω_a _*per filament at which cytosolic NPFs bind. In addition, we assume a cooperative effect among bound NPFs, namely, that bound NPFs favor the binding of additional NPFs, see Figure 4d. We refrain from suggesting a possible molecular picture underlying this effect and simply note that some cooperative effect is necessary to spontaneously generate waves. Altogether, we arrive at the following dynamic equations:

Also the dynamics of the NPFs is governed by continuity equations. The transport of NPFs is purely diffusive. This holds also for bound NPFs as they stay attached to a specific subunit of a filament and as treadmilling does not move filament subunits. Note, however, that the system behavior does not change qualitatively if NPFs are transported with the filaments, as could be the case in presence of molecular motors. For bound NPFs we assume a constant unbinding rate *ω_d_*, see Figure 4e. The attachment dynamics of NPFs to filaments is more involved. First of all there is a base rate *ω_a _*per filament at which cytosolic NPFs bind. In addition, we assume a cooperative effect among bound NPFs, namely, that bound NPFs favor the binding of additional NPFs, see Figure 4d. We refrain from suggesting a possible molecular picture underlying this effect and simply note that some cooperative effect is necessary to spontaneously generate waves. Altogether, we arrive at the following dynamic equations:

(36) $$\frac{\partial }{\partial t}n^{+}=D\frac{\partial^{2}}{\partial x^{2}}n^{+}+\omega_{a}c^{+}\left(1+\omega_{1}n^{{+}^{2}}\right)n_{c}-\omega_{d}n^{+}$$

(37) $$\frac{\partial }{\partial t}n^{-}=D\frac{\partial^{2}}{\partial x^{2}}n^{-}+\omega_{a}c^{-}\left(1+\omega_{1}n^{{-}^{2}}\right)n_{c}-\omega_{d}n^{-}$$

(38) $$\frac{\partial }{\partial t}n_{c}=D_{c}\frac{\partial^{2}}{\partial x^{2}}n_{c}-\omega_{a}\left(c^{+}\left(1+\omega_{1}n^{{+}^{2}}\right)+c^{-}\left(1+\omega_{1}n^{{-}^{2}}\right)\right)n_{c}+\omega_{d}\left(n^{+}+n^{-}\right).$$

In this expression, *D*_c _is the diffusion constant for cytosolic NPFs and *ω*_1 _is a measure of the cooperative effect on NPF attachment. We have assumed that NPFs are neither generated nor do they degrade.

**Your turn: **Show that the total number of NPFs is conserved.

This completes our description of treadmilling filaments. There are now several ways to analyze the above equations. An analytic solution of such coupled non-linear partial differential equations is usually not possible and one thus relies on numerical solutions. Before describing one method to numerically solve the equations, let us briefly comment on a powerful method to get an overview about the parameter values for which we might expect interesting behavior.

### Linear stability analysis

This method starts by realizing that often, there is one rather simple solution to a continuum theory. Indeed, as is the case for the above equations, there is a spatially homogenous steady state, which is obtained by setting all derivatives equal to zero. This leaves us with an algebraic set of equations, which can be solved numerically. The corresponding homogenous densities are denoted by $c_{0}^{+}$, $c_{0}^{-}$, $n_{0}^{+}$, $n_{0}^{-}$, and *n*_c,0_

**Your turn: **Derive the equations for the homogenous steady state of Eqs. (30), (31), and (36)-(38).

A real system is permanently subject to fluctuations. What happens to the spatially homogenous state if it is slightly perturbed? To study this question, we can write the distributions in the form $c^{+}\left(x\right)=c_{0}^{+}+\delta c^{+}\left(x\right)$ and analogously for *c^-^*, *n*^+^, *n^-^*, and *n*_c_. Inserting these expressions into the dynamic equations and keeping only terms in linear order in the perturbation, we arrive at a set of linear differential equations.

**Your turn: **Derive the differential equations up to linear order in the perturbations *δc*^+^, *δc^-^*, *δn*^+^, *δn^-^*, and *δn*_i_. What happens to the zeroth order terms?

The linearized equations are easier to solve than the nonlinear dynamic equations, sometimes even analytically. We do not want to do this explicitly here, as it requires some advanced mathematical techniques. Suffice it here to state that the linear stability analysis not only allows to identify interesting regions in parameter space, but often even yields some properties of the new state. In particular, it can indicate, whether the states that the system assumes under conditions of an unstable homogenous state is stationary or oscillatory. However, only a nonlinear analysis can give the real answer. To this end one usually solves the dynamic equations numerically.

### Numerical solution

There are many sophisticated methods to numerically solve non-linear partial differential equations. We will briefly introduce one very simple method that makes direct contact to the considerations in the mathematical interlude. While it may be slow, it works in a remarkably large number of cases.

In this numerical scheme, we discretize space and time - to some end we are thus going to reverse the continuum limit. Let us start by discretizing time. We only consider the density at discrete time points *t *= *k*Δ*t*, where Δ*t *is the discretization length and *k *some integer. Then the derivative with respect to time is replaced by

(39) $$\frac{d}{dt}c\left(t\right)\approx \frac{c\left(\left(k+1\right)\Delta t\right)-c\left(k\Delta t\right)}{\Delta t}.$$

Thus, given the time evolution equation

(40) $$\frac{d}{dt}c=f\left(c\left(t\right),t\right),$$

we can obtain *c*((*k *+ 1)Δ*t*) from *c*(*k*Δ*t*) by writing

(41) c((k+1)Δt)=c(kΔt)+Δtf(c(kΔt),kΔt).

In addition to time, the filament and nucleator densities also depend on space. Let *c_i_*(*k*Δ*t*) be the density at position *i*Δ*x*, where Δ*x *is the spatial discretization length and *i *an integer with 0 <*i *≤ *L*/Δ*x *and consider the equation

(42) $$\frac{\partial }{\partial t}c\left(x,t\right)=-\frac{\partial }{\partial x}j\left(x,t\right).$$

An approximate expression for *c_i_*((*k *+ 1)Δ*t*) is then obtained from

(43) $$c_{i}\left(\left(k+1\right)\Delta t\right)=c_{i}\left(k\Delta t\right)+\Delta t\left(j_{i}\left(k\Delta t\right)-j_{i+1}\left(k\Delta t\right)\right)/\Delta x.$$

In this expression *j_i _*denotes the current from site *i - *1 to site *i*. Since *c_i_*Δ*x *is number of filaments in the bin *i*, this equation is the formal analog of Figure 2a.

The discretized expressions for *j *= *vc *is

(44) $$j_{i}=vc_{i-1}$$

if *v *> 0 and

(45) $$j_{i}=vc_{i}$$

if *v *< 0. Inserting this into Eq. (43), we get

(46) $$c_{i}\left(\left(k+1\right)\Delta t\right)=c_{i}\left(k\Delta t\right)+\Delta t\left(vc_{i-1}-vc_{i}\right)/\Delta x,$$

where we have considered the case *v *> 0. Since *c_i _*is a particle density, it must always be positive. From this condition, we get 1 *- v*Δ*t/*Δ*x *> 0 as a sufficient condition. That is, the values of Δ*t *and Δ*x *are not completely independent! Finally, we provide the expression for the diffusion current

(47) $$j_{i}=D\left(c_{i-\text{1}}-c_{i}\right)/\Delta x.$$

**Your turn: **What is the condition on Δ*x *and Δ*t *resulting from this current?

**Figure 5:**
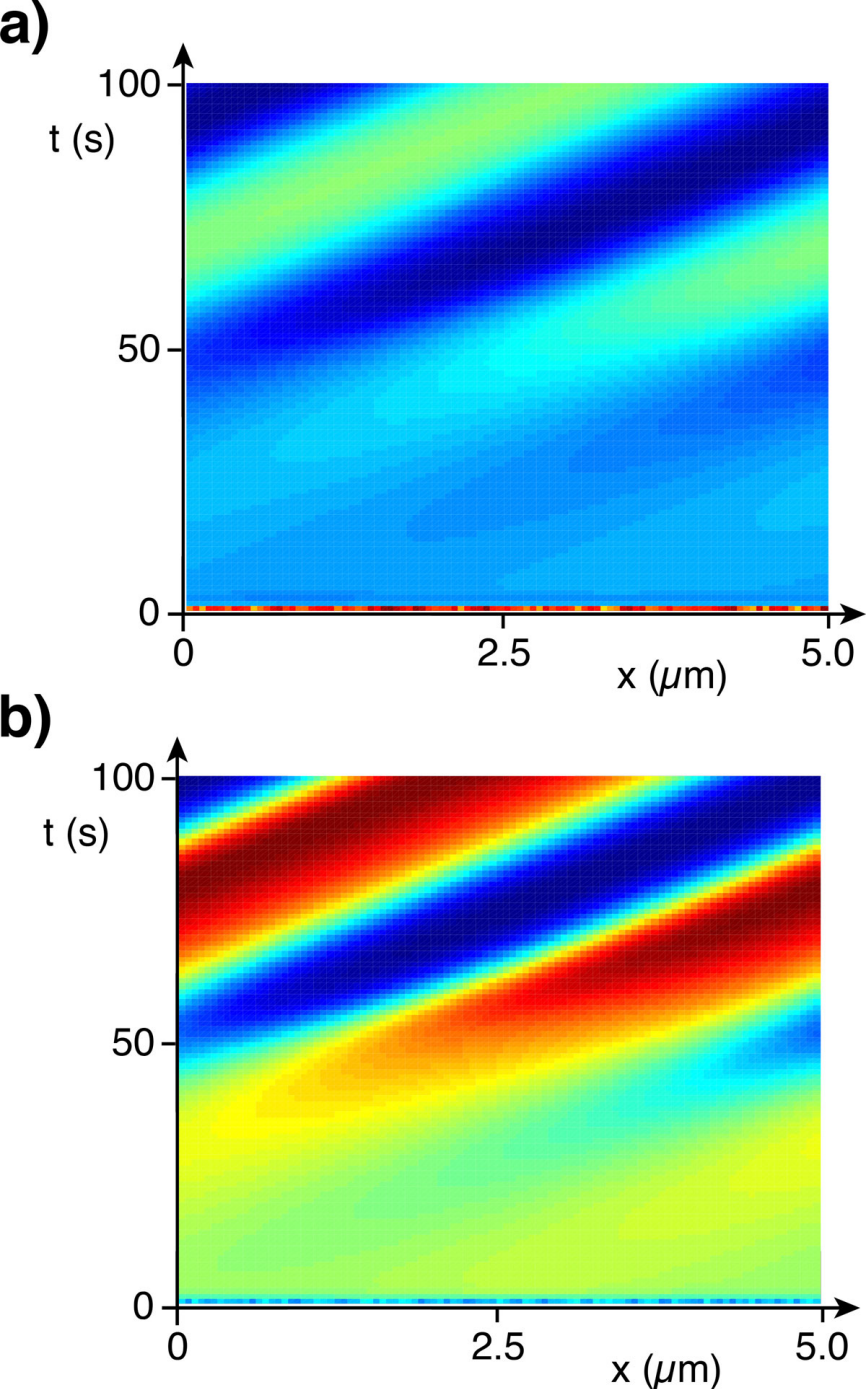
**Spontaneous cytoskeletal wave**. Numerical solution to the dynamic equations (30)-(38). **a) **Filament density. **b) **Density of filament-bound NPFs. Warmer colors indicate higher densities. Parameter values are *v *= 0.1 *μ*m/s, *D *= 0.01 *μ*m^2^/s, *D*_c _= 0.1 *μ*m^2^/s, *ν*= 0.1s^-1^, *ν*_*d *_= 0.1s^-1^, *ω_d _*= 0.1s^-1^, *ω_a _*= 0.01 *μ*m/s, and *ω*_1 _= 100 *μ*m^2^.

In Figure 5 we present space-time plots of the total NPF and filament densities in the case of an unstable homogenous distribution. In this case, the system indeed self-organizes into a traveling wave: Starting from a random initial condition, the filaments and NPFs form a distribution that moves at constant velocity.

**Figure 6:**
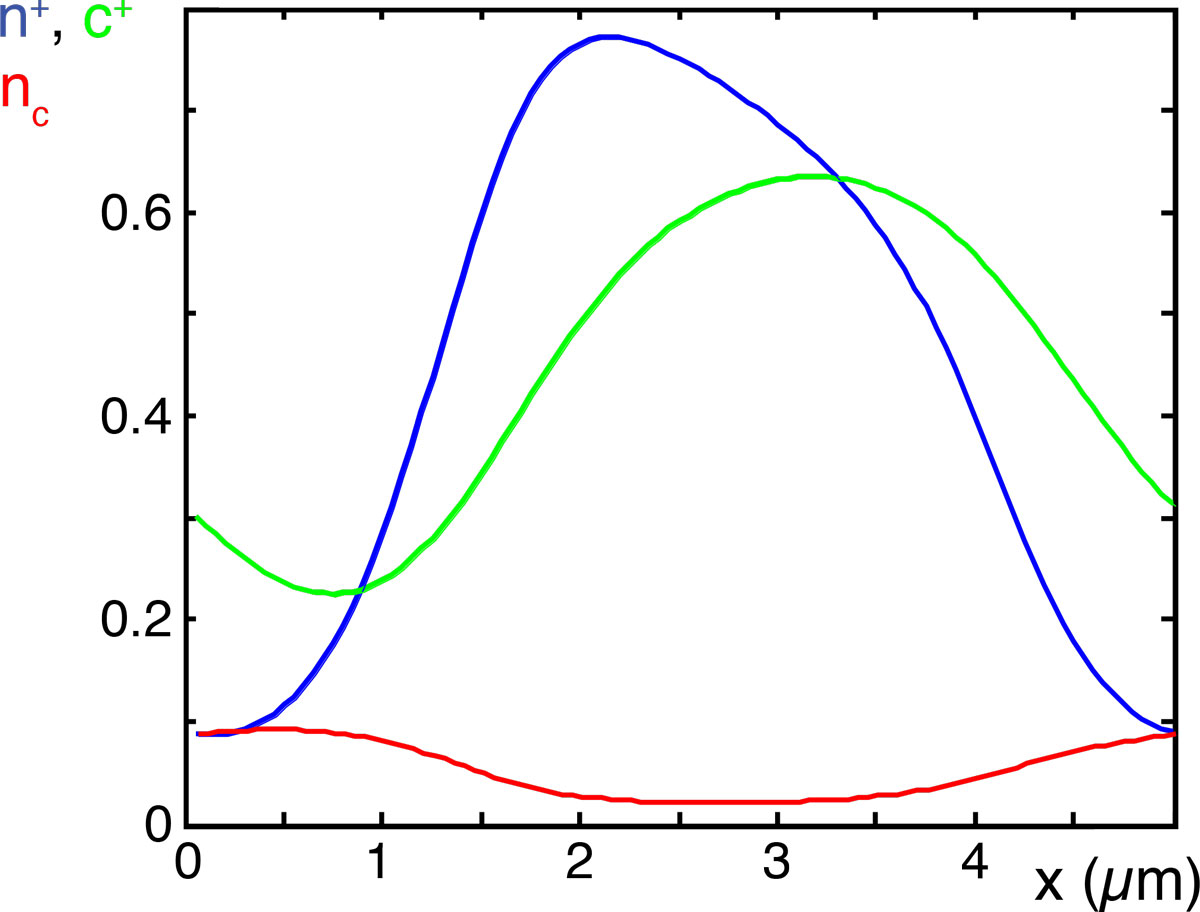
**Filament and NPF densities in a wave**. Densities *c*^+^, *n*^+^, *n*_c _of plus-filaments (green), plus-NPFs (blue), and cytosolic NPFs (red) obtained from a numerical solution to the dynamic equations (30)-(38). Densities correspond to the state at time *t *= 100s in Figure 3. The densities of *c^- ^*and *n^- ^*are too small (< 10^-4^) to be seen in the graph.

We can obtain additional insight into this state by plotting the various densities, see Figure 6. It turns out that there are practically only filaments of one orientation, while the density of filaments of the opposite orientation is negligible. Similarly, this holds for the corresponding filament-bound NPFs. Depending on the initial state, either one of the orientations will win and a wave either moving to the right or to the left will appear. Note, that we have not included any directional motion for the NPFs into our description. The apparent motion of the corresponding densities is a result of diffusion as well as binding to and unbinding from filaments: The peaks of the respective distributions of the NPFs and the filaments are shifted with the NPFs lagging behind, such that NPFs bind preferentially ahead of its maximum density.

## Conclusion

In this tutorial, we have given an introduction into continuum theories as a tool for analyzing cytoskeletal processes. We gave one example of a description that was derived from a discrete (microscopic) picture, and one example that was developed entirely within the frame of the continuum approach and was based on the continuity equation. Let us mention, that not all relevant quantities obey the continuity equation. If you consider, for example, a cytoskeletal network in two or three spatial dimensions, then one might need to take into account that, on large scales, the network can present orientational order. Since the filament orientation is not a conserved quantity, its dynamics is not given by the continuity equation. To derive dynamic equations for such fields one either relies on microscopic theories or on symmetry considerations.

In the examples we discussed, we assumed a deterministic evolution of the system. In reality, the molecular processes underlying cellular processes are stochastic. In general, it is not an easy task to obtain the correct order of magnitude of stochastic effects. As long as one is only interested in qualitative behavior, it is often sufficient to capture molecular noise by effective diffusion terms.

Probably a more serious point is that we neglected in our examples mechanical forces altogether. However, in cells and often also in reconstituted systems forces play an important role. These can be incorporated into continuum theories by accounting also for momentum conservation in addition to matter conservation. Sources and sinks are in this case given by external forces, while the momentum flux density is given by the stress tensor. In addition to viscous or visco-elastic properties, the stress tensor also accounts for the stress generated by active processes [4]. In contrast, energy conservation is usually not an issue as cellular systems are embedded in a heat reservoir which maintains the system at constant temperature.

In addition to the formal aspects of the descriptions developed above, let us comment on the biological significance of the processes considered. There is at least indirect experimental evidence for a feedback between the activity of nucleation promoting factors and actin filaments in neutrophils [14], although the molecular players involved are probably not completely known and mechanisms remain to be identified. As mentioned already above, the assembly dynamics of cytoskeletal filaments was also greatly simplified to not distract the reader from the focus of this tutorial. Indeed, neither the assembly nor the disassembly rates of filaments are expected to remain constant in time as typically they depend effectively on the filament length [19]. The assumption of constant assembly and disassembly rates prompted us to also assume a rate at which filaments spontaneously disintegrate. Although this is again unlikely to be the case in living cells, the qualitative behavior of the artificial system is comparable to specific situations in more complicated scenarios [16]. A thorough discussion of realistic applications of continuum theories to cytoskeletal or other-cell-biological systems goes beyond the aim of the present tutorial.

In summary, continuum descriptions provide powerful tools to analyze cellular processes. In a number of cases, they have provided valuable information about biological and, in particular, cellular structures and processes. It will be exciting to see, whether they can also play a role in designing new therapeutic approaches.

## Competing interests

The author declares that they have no competing interests.
